# Provisional Mortality Data — United States, 2022

**DOI:** 10.15585/mmwr.mm7218a3

**Published:** 2023-05-05

**Authors:** Farida B. Ahmad, Jodi A. Cisewski, Jiaquan Xu, Robert N. Anderson

**Affiliations:** 1 National Center for Health Statistics, CDC.

The National Center for Health Statistics’ (NCHS) National Vital Statistics System (NVSS) collects and reports annual mortality statistics using U.S. death certificate data. Because of the time needed to investigate certain causes of death and to process and review death data, final annual mortality data for a given year are typically released 11 months after the end of the calendar year. Provisional data, which are based on the current flow of death certificate data to NCHS, provide an early estimate of deaths, before the release of final data. NVSS routinely releases provisional mortality data for all causes of death and for deaths associated with COVID-19.* This report is an overview of provisional U.S. mortality data for 2022, including a comparison with 2021 death rates. In 2022, approximately 3,273,705 deaths^†^ occurred in the United States. The estimated 2022 age-adjusted death rate decreased by 5.3%, from 879.7 per 100,000 persons in 2021 to 832.8. COVID-19 was reported as the underlying cause or a contributing cause in an estimated 244,986 (7.5%) of those deaths (61.3 deaths per 100,000). The highest overall death rates by age, race and ethnicity, and sex occurred among persons who were aged ≥85 years, non-Hispanic American Indian or Alaska Native (AI/AN), non-Hispanic Black or African American (Black), and male. In 2022, the four leading causes of death were heart disease, cancer, unintentional injuries, and COVID-19. Provisional death estimates provide an early indication of shifts in mortality trends and can guide public health policies and interventions aimed at reducing mortality, including deaths directly or indirectly associated with the COVID-19 pandemic.

NCHS analyzed provisional NVSS death certificate data for deaths occurring among U.S. residents in the United States during January–December 2022. Deaths among residents of U.S. territories and foreign countries were excluded. The number and rates of overall deaths and COVID-19–associated deaths were tabulated by age, sex, and race and ethnicity (categorized as AI/AN, non-Hispanic Asian [Asian], Black, non-Hispanic Native Hawaiian or other Pacific Islander, non-Hispanic White [White], non-Hispanic persons of more than one race [multiracial], unknown, or Hispanic or Latino [Hispanic]). NCHS coded the causes of death according to the *International Classification of Diseases, Tenth Revision*, which details disease classification and the designation of underlying cause of death (*1*,*2*). COVID-19–associated death counts and rates include deaths for which COVID-19 was listed on the death certificate as an underlying or contributing cause of death.^§^ COVID-19 was the underlying cause for approximately 76% (186,702) of COVID-19–associated deaths in 2022 (*3*). Leading causes of death were ranked by counts based on underlying cause of death (*4*). Age was unknown for 101 (<0.01%) decedents, and race and ethnicity were unknown for 10,086 (0.31%). The trends in deaths during the year were determined by calculating the number of deaths for each week from all causes and from COVID-19. Age-adjusted death rates were calculated for deaths overall and by sex and race and ethnicity. Crude death rates were calculated by age. The population data used to calculate death rates are July 1, 2021 estimates based on the Blended Base produced by the U.S. Census Bureau (*5*,*6*). Unless otherwise specified, rate comparisons in the text are statistically significant (p<0.05).

In 2022, approximately 3,273,705 deaths occurred in the United States (Table). The age-adjusted rate, 832.8 deaths per 100,000 standard population, represented a decrease of 5.3% from 879.7 in 2021 (*7*). The total number of deaths peaked during the weeks ending January 22, 2022 (85,405 deaths) and December 31, 2022 (69,664) (Figure 1). In 2022, total death rates were lowest among persons aged 5–14 years (14.8 per 100,000) and highest among persons aged ≥85 years (15,605.2), similar to patterns in 2021 (Table). Overall death rates decreased for all age groups ≥15 years from 2021 to 2022, while the death rate increased from 25.0 to 26.9 per 100,000 among persons aged 1–4 years during the same period. The changes in overall death rates for age groups <1 year and 5–14 years were not significant. Age-adjusted death rates were higher among males compared with females in 2021 (1,048.0 and 733.3, respectively) and 2022 (984.8 and 700.9, respectively); however, from 2021 to 2022, the death rates decreased 6.0% among males and 4.4% among females.

**TABLE:** Provisional* number and rate of total deaths and COVID-19–associated deaths, by demographic characteristic — National Vital Statistics System, United States, 2021–2022

Characteristic	No. of deaths (rate^†^)			
	2021		2022	
	Total	COVID-19–associated^§^	Total	COVID-19–associated^§^
**Total**	**3,464,231 (879.7)**	**462,193 (115.6)**	**3,273,705 (832.8)**	**244,986 (61.3)**
**Age group, yrs**				
<1	**19,920 (558.8)**	167 (4.7)	20,238 (567.8)	231 (6.5)
1–4	**3,816 (25.0)**	66 (0.4)	4,107 (26.9)	152 (1.0)
5–14	**5,975 (14.3)**	185 (0.4)	6,193 (14.8)	203 (0.5)
15–24	**38,307 (88.9)**	1,652 (3.8)	35,064 (81.4)	641 (1.5)
25–34	**82,274 (180.8)**	7,033 (15.5)	74,025 (162.7)	2,376 (5.2)
35–44	**124,939 (287.9)**	17,412 (40.1)	111,151 (256.1)	5,183 (11.9)
45–54	**216,037 (531.0)**	39,360 (96.7)	182,689 (449.0)	12,169 (29.9)
55–64	**478,171 (1,117.1)**	79,199 (185.0)	416,393 (972.8)	30,526 (71.3)
65–74	**724,266 (2,151.3)**	111,412 (330.9)	667,308 (1,982.1)	53,228 (158.1)
75–84	**829,653 (5,119.4)**	110,536 (682.1)	823,908 (5,083.9)	67,116 (414.1)
≥85	**940,780 (15,743.3)**	95,168 (1,592.6)	932,528 (15,605.2)	73,157 (1,224.2)
Unknown	**93 (—)**	3 (—)	101 (—)	4 (—)
**Sex**				
Female	**1,626,123 (733.3)**	202,687 (91.8)	1,558,144 (700.9)	112,287 (49.8)
Male	**1,838,108 (1,048.0)**	259,506 (144.5)	1,715,561 (984.8)	132,699 (76.3)
**Race and ethnicity**				
AI/AN, NH	**26,972 (1,109.2)**	5,053 (201.8)	23,440 (973.3)	2,115 (86.8)
Asian, NH	**92,432 (461.7)**	13,707 (66.6)	88,963 (447.2)	6,786 (34.1)
Black or African American, NH	**449,764 (1,118.0)**	61,959 (151.4)	410,126 (1,028.0)	28,695 (72.9)
NH/OPI, NH	**5,223 (924.3)**	1,175 (200.9)	4,590 (824.8)	378 (67.8)
White, NH	**2,548,809 (893.9)**	304,586 (105.0)	2,444,427 (855.4)	180,212 (61.2)
Hispanic or Latino	**315,664 (724.7)**	72,910 (161.7)	275,254 (643.4)	25,076 (60.9)
Multiracial, NH	**17,316 (406.0)**	2,018 (50.7)	16,819 (394.2)	1,045 (26.7)
Unknown	**8,051 (—)**	785 (—)	10,086 (—)	679 (—)

**Abbreviations:** AI/AN = American Indian or Alaska Native; NH = non-Hispanic; NH/OPI = Native Hawaiian or other Pacific Islander.

* National Vital Statistics System provisional data for 2022 are incomplete. Data for 2021 are final. These data exclude deaths that occurred in the United States among residents of U.S. territories and foreign countries.

^†^ Deaths per 100,000 standard population. Age-adjusted death rates are provided overall and by sex and race and ethnicity.

^§^ Deaths of persons coded to *International Classification of Diseases, Tenth Revision* (code U07.1), with COVID-19 as an underlying or contributing cause of death.

**FIGURE 1:**
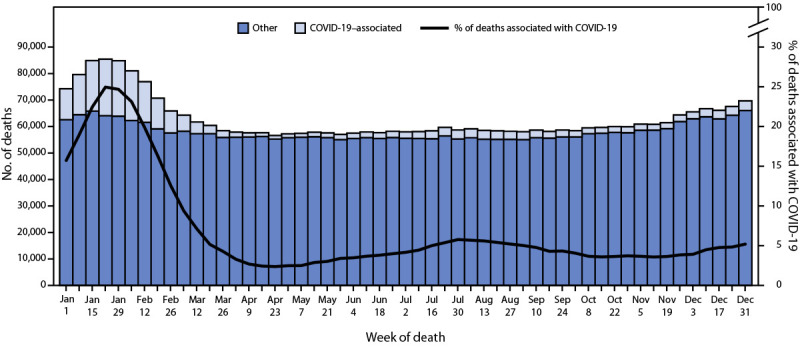
Provisional* number of COVID-19–associated deaths^†^ and other deaths and percentage of COVID-19–associated deaths, by week of death — National Vital Statistics System, United States, 2022 * National Vital Statistics System provisional data for 2022 are incomplete. Data from December 2022 are less complete because of reporting lags. Data for 2021 are final. These data exclude deaths that occurred in the United States among residents of U.S. territories and foreign countries. ^† ^Deaths of persons coded to *International Classification of Diseases*, *Tenth Revision* (code U07.1), with COVID-19 as an underlying or contributing cause of death.

During 2022, COVID-19 was listed as the underlying or contributing cause of 244,986 deaths (61.3 per 100,000), a 47% decrease from 462,193 deaths (115.6 per 100,000) in 2021. The COVID-19–associated death rate decreased from 2021 to 2022 among age groups ≥15 years, and the rate increased for all age groups <15 years. As with deaths overall, the age-adjusted COVID-19–associated death rate among males (76.3) was higher compared with that among females (49.8).

Age-adjusted death rates differed by race and ethnicity and decreased for all groups from 2021 to 2022. Overall age-adjusted death rates were lowest among multiracial (394.2) and Asian persons (447.2) and highest among Black (1,028.0) and AI/AN (973.3) persons. COVID-19–associated death rates declined from 2021 to 2022 for all racial and ethnic groups.

During 2022, the three leading causes of death were heart disease (699,659 deaths), cancer (607,790), and unintentional injury (218,064) (Figure 2).^¶^ COVID-19, listed as the underlying cause for 186,702 deaths during 2022, ranked as the fourth leading underlying cause of death. COVID-19 was the underlying cause for 5.7% of all deaths in 2022, decreasing from 12.0% (416,893 deaths) in 2021. Heart disease and cancer deaths increased in 2022 compared with 2021 (accounting for 695,547 and 605,213, deaths respectively), while deaths associated with COVID-19 decreased.

**FIGURE 2:**
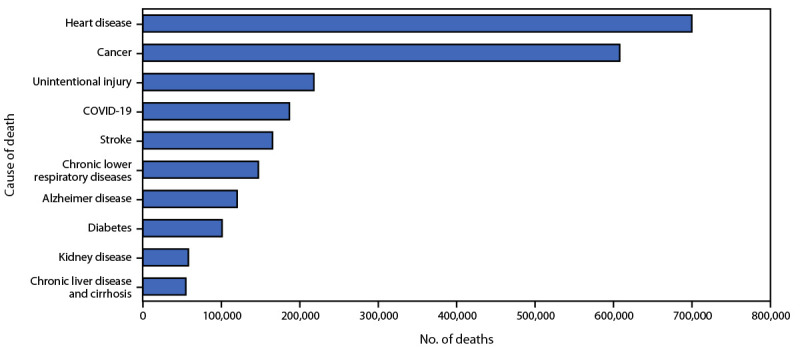
Leading underlying causes of death*^,^^†^— National Vital Statistics System, United States, 2022 * Data are provisional; National Vital Statistics System provisional data are incomplete, and data from December are less complete because of reporting lags. Deaths that occurred in the United States among residents of U.S. territories and foreign countries were excluded. ^†^ Deaths are ranked by number of deaths per underlying cause of death.

## Discussion

During January–December 2022, the estimated 2022 U.S. age-adjusted death rate decreased by 5.3% to 832.8 per 100,000 persons, from 879.7 in 2021. Overall death rates were highest among males, older adults, and Black persons. The highest weekly numbers of overall deaths and COVID-19–associated deaths occurred during January and December. The three leading causes of death in 2022 were heart disease, cancer, and unintentional injury. COVID-19, the third leading cause of death in 2021, fell to fourth place in 2022 because of the large decrease in COVID-19–associated deaths compared with those in 2021 (*7*). The number of deaths caused by unintentional injury, largely driven by a high number of drug overdose deaths, remained high in 2022 compared with 2021 (*8*).

Overall death rates and COVID-19–associated death rates decreased from 2021 to 2022 for most demographic groups, with the exception of certain age groups. COVID-19–associated death rates increased for all persons aged <15 years. Although the overall and COVID-19–associated death rates decreased for persons aged ≥85 years from 2021 to 2022, rates remained higher for this group compared with all other age groups. In addition, although overall and COVID-19–associated death rates decreased among all racial and ethnic groups, age-adjusted total and COVID-19–associated death rates remained high for Black and AI/AN persons compared with other groups. The current report did not examine death rates for causes of death other than COVID-19; however, available provisional data from the CDC WONDER platform indicate death rate patterns for leading causes of death (*9*). The age-adjusted rate of heart disease deaths increased for the third straight year since 2020. While the age-adjusted rate of cancer deaths had declined steadily during 1999–2020, the cancer death rate increased in 2021 and 2022. Further analysis of provisional data might offer additional insights into demographic patterns of leading causes of death.

The findings in this report are subject to at least three limitations. First, data are provisional, and numbers and rates might change as additional information is received. Described changes in mortality trends might be underestimates. Second, timeliness of death certificate submission can vary by jurisdiction. As a result, the national distribution of deaths might be affected by the distribution of deaths reported from jurisdictions reporting later, which might differ from those in the United States overall. Finally, potential exists for misclassification of certain categories of race (i.e., AI/AN and Asian) and Hispanic ethnicity reported on death certificates (*10*). Thus, death rates for some groups might be under- or overestimated.

This report provides an overview of provisional mortality in the United States during 2022. Provisional death estimates can offer researchers and policymakers an early signal about shifts in mortality trends and provide actionable information sooner than do the final mortality data, which are released approximately 11 months after the end of the data year. These data can guide public health policies and interventions aimed at reducing mortality directly or indirectly associated with the COVID-19 pandemic and among persons most affected, including persons who are older, male, or from members of certain racial and ethnic minority groups.

**Summary**

**What is already known about this topic?**

More than 3.2 million persons died in the United States during January–December 2022.

**What is added by this report?**

The overall age-adjusted U.S. death rate decreased by 5.3% from 2021 to 2022. Overall death rates and COVID-19–associated death rates were highest among non-Hispanic Black or African American persons and non-Hispanic American Indian or Alaska Native persons.

**What are the implications for public health practice?**

Provisional death estimates provide an early signal about shifts in mortality trends. Timely and actionable data can guide public health policies and interventions for populations experiencing higher mortality.

## Footnotes

* NVSS provisional mortality data are available at CDC’s Provisional WONDER platform (https://wonder.cdc.gov). NVSS COVID-19 surveillance data are available online. https://www.cdc.gov/nchs/nvss/deaths.htm

† Based on death records received and processed as of April 2, 2023, for deaths occurring in the United States among U.S. residents. Data included in this analysis include >99% of deaths occurring in 2022.

§ The underlying cause of death is the disease or injury that initiated the chain of morbid events leading directly to death.

¶ In this report, the number of deaths caused by unintentional injury was tabulated using the internal NVSS mortality database and might not match publicly available provisional counts for deaths for unintentional injury because provisional data for injury-related causes of death are publicly released with a lag of 6 months from the date of death. This delay accounts for the additional time typically needed to report injury-related death certificates, and the final 2022 death count might be higher than noted in this report.
